# Predicting Outcome and Therapy Response in mCRC Patients Using an Indirect Method for CTCs Detection by a Multigene Expression Panel: A Multicentric Prospective Validation Study

**DOI:** 10.3390/ijms18061265

**Published:** 2017-06-13

**Authors:** Yolanda Vidal Insua, Juan De La Cámara, Elena Brozos Vázquez, Ana Fernández, Francisca Vázquez Rivera, Mª José Villanueva Silva, Jorge Barbazán, Laura Muinelo-Romay, Sonia Candamio Folgar, Alicia Abalo, Rafael López-López, Miguel Abal, Lorena Alonso-Alconada

**Affiliations:** 1 Translational Medical Oncology, Health Research Institute of Santiago de Compostela (IDIS), University Hospital of Santiago de Compostela, SERGAS Group, Trav. Choupana s/n, 15706 Santiago de Compostela, Spain; Yolanda.Vidal.Insua@sergas.es (Y.V.I.); elenambrozosv@hotmail.com (E.B.V.); Francisca.Vazquez.Rivera@sergas.es (F.V.R.); jorgebarbazan@gmail.com (J.B.); lmuirom@gmail.com (L.M.-R.); soniasigueiro@yahoo.es (S.C.F.); alicia.abalo.pineiro@sergas.es (A.A.); Rafael.lopez.lopez@sergas.es (R.L.-L.); miguel.abal.posada@sergas.es (M.A.); 2 Arquitecto Macide Hospital, SERGAS Group, Av. Residencia s/n, 15405 Ferrol, Spain; juan.cruz.de.la.camara.gomez@sergas.es; 3 University Hospital of Ourense, SERGAS Group, Ramon Puga 54, 32005 Ourense, Spain; afm1003@hotmail.com; 4 University Hospital of Vigo, SERGAS Group, Pizarro 22, 36204 Vigo, Spain; maria.jose.villanueva.silva@sergas.es; 5 Liquid Biopsy Analysis Unit, Health Research Institute of Santiago de Compostela (IDIS), University Hospital of Santiago de Compostela, SERGAS Group, Trav. Choupana s/n, 15706 Santiago de Compostela, Spain

**Keywords:** metastatic colorectal cancer, circulating tumor cells, biomarkers, therapy response

## Abstract

Colorectal cancer (CRC) is one of the major causes of cancer-related deaths. Early detection of tumor relapse is crucial for determining the most appropriate therapeutic management. In clinical practice, computed tomography (CT) is routinely used, but small tumor changes are difficult to visualize, and reliable blood-based prognostic and monitoring biomarkers are urgently needed. The aim of this study was to prospectively validate a gene expression panel (composed of *GAPDH*, *VIL1*, *CLU*, *TIMP1*, *TLN1*, *LOXL3* and *ZEB2*) for detecting circulating tumor cells (CTCs) as prognostic and predictive tool in blood samples from 94 metastatic CRC (mCRC) patients. Patients with higher gene panel expression before treatment had a reduced progression-free survival (PFS) and overall-survival (OS) rates compared with patients with low expression (*p* = 0.003 and *p* ≤ 0.001, respectively). Patients with increased expression of CTCs markers during treatment presented PFS and OS times of 8.95 and 11.74 months, respectively, compared with 14.41 and 24.7 for patients presenting decreased expression (PFS; *p* = 0.020; OS; *p* ≤ 0.001). Patients classified as non-responders by CTCs with treatment, but classified as responders by CT scan, showed significantly shorter survival times (PFS: 8.53 vs. 11.70; OS: 10.37 vs. 24.13; months). In conclusion, our CTCs detection panel demonstrated efficacy for early treatment response assessment in mCRC patients, and with increased reliability compared to CT scan.

## 1. Introduction

Colorectal cancer (CRC) is one of the most common cancers worldwide, and it is the third cause of cancer deaths in western countries [1]. About 25% of cases are diagnosed in an advanced stage and up to 40% of non-metastatic patients at diagnosis end up developing distant metastasis [2]. Survival in early stages reaches an 80–95% rate, dropping substantially to ≈8% in metastatic patients. Treatment of metastatic CRC (mCRC) is generally palliative, with the goal of prolonging overall survival (OS) and maintaining quality of life. Standard antineoplastic drugs are fluoropyrimidines (fluouracil or capecitabine), either in monotherapy or combined with oxaliplatin/irinotecan and/or targeted drugs (anti-vascular endothelial growth factor receptor (anti-VEGFR) like bevacizumab or aflibercept, and anti-epidermal growth factor receptor (anti-EGFR) like cetuximab or panitumumab, based on Rat Sarcoma (RAS) oncogene mutation status). These treatments achieve an increase in OS rates from six to 24 months [3,4,5,6]. Nevertheless, and despite advances in the treatment of mCRC, numerous patients do not respond to therapy and suffer toxicity-related side effects.

Monitoring of patients represents a critical issue in the management of therapeutic strategies. Currently, the gold standard methodology for evaluating therapy response is computed tomography (CT). This image-based approach provides reliable information about location and tumor burden, enabling the clinical community to use of a standardized evaluation for treatment response by response evaluation criteria in solid tumors (RECIST) [7]. These criteria allow a high specific response, and are useful when assessing the efficacy of therapeutic agents that operate via a tumor-shrinkage mechanism. However, different studies suggest that when the way of action of a therapy is mediated by other mechanisms, such as with immunotherapy or molecular targeted treatments, the tumor size is not directly correlated with the clinical outcome [8]. Despite undeniable CT utility, occult lesions may be difficult to visualize with this methodology, which cannot be repeatedly applied as it imposes risks for the patient, due to repeated ionic radiation and allergenic contrast exposure. In patients with mCRC, a rapid availability of therapy response data determines the quantity and quality of life, and this still remains a challenge in oncology, which has not yet progressed with imaging techniques or currently available molecular markers like carcinoembryonic antigen (CEA).

In the last few years, clinical evidence has indicated a key role for circulating tumor cells (CTCs) dissemination from primary tumors in the generation of distant metastasis [9]. CTCs detection and quantification have shown a prognostic value in different tumor types, including CRC, which is why the CellSearch System (Veridex, Janssen Diagnostics, South Raritan, NJ, USA) was approved by the American Food and Drug Administration (FDA) for CTCs enumeration in mCRC patients. Using this system, it has been described that mCRC patients who present three or more CTCs per 7.5 mL of blood showed poor patient outcome at baseline, e.g., before treatment [10,11]. More interestingly, CTCs have also been proposed as a treatment response predictive biomarker, mainly in breast cancer. These findings are being explored in other tumors like prostate, lung or colorectal cancers [12,13,14].

To date, limited studies have shown the utility of analyzing changes in the number of CTCs or in their gene expression profiles, during treatment in mCRC patients. In a previous work from our group, we described the value of CTCs analysis by a multimarker gene expression panel (PrediCTC), which included intestine-specific and epithelial to mesenchymal transition (EMT) transcripts [15]. CTCs were immunoisolated from peripheral blood samples before and at four and 16 weeks after treatment onset, from 50 mCRC patients receiving first-line antineoplastic treatment. Patients with increased expression of PrediCTC markers along treatment had shorter progression-free survival (PFS) and OS times, compared to patients where marker expression decreased. Using this multimarker panel, we could identify patients who were not benefiting from the therapy, earlier than routine CT scans, and from a minimally invasive liquid biopsy. Of note, PrediCTC identified therapy-refractory patients who were not detected by standard image techniques, demonstrating improved sensitivity.

The novelty of this new work includes the clinical validation of PrediCTC in a prospective multicentric study, and the assessment of technology efficacy compared to standard CT scans, with 94 mCRC patients receiving antineoplastic therapy as part of first-line treatments. This allowed limitations such as single center and low sample size to be addressed, and to demonstrate PrediCTC as an efficient therapy response prognostic tool.

## 2. Results

### 2.1. Prognostic Value of Circulating Tumor Cells (CTCs) Individual Markers

A total of 94 patients with mCRC were included in this multicentric prospective study for the validation of PrediCTC as a new technology based on a multimarker gene-panel in circulating tumor cells (CTCs) for the rapid assessment of therapy response. PrediCTC is based on a combination of seven CTCs biomarkers previously described by our group, which have demonstrated both diagnostic and prognostic value in mCRC patients. These markers included *TIMP1* and *CLU*, selected in a previous global gene expression profiling study developed in our group [16], *VIL1* as an epithelial marker, and *LOXL3* and *ZEB2* as transcription factors involved in EMT and associated with metastasis dissemination. We also included *TLN1* as a gene involved in CTCs extravasation during metastatic dissemination [17]. *GAPDH* was also included as a marker of global cellularity. Finally, *PTPRC* was used as an indicator of unspecific cell isolation, and the expression levels of all other genes were normalized to *PTPRC* expression levels, as previously reported [18].

The expression levels for each CTCs individual marker are shown in Table S1 as well as the cutoff value for each biomarker at baseline, and at four- and 16-week time points. As shown in Table 1, all CTCs-markers that presented an expression above the cutoff (“high CTC” content) showed a direct correlation with shorter overall survival (OS) rates, both when analyzed at baseline and at four weeks of treatment. Similarly, patients presenting CTCs gene expression levels (*VIL1*, *CLU*, *TIMP* and *LOXL3*) above cut-off at baseline showed a significant reduction in PFS. The same was observed for patients with *GAPDH*, *LOXL3* and *ZEB2* gene expression levels above cut-off at four weeks after treatment onset (Table 1).

### 2.2. PrediCTC Prognostic Value

**Figure 1:**
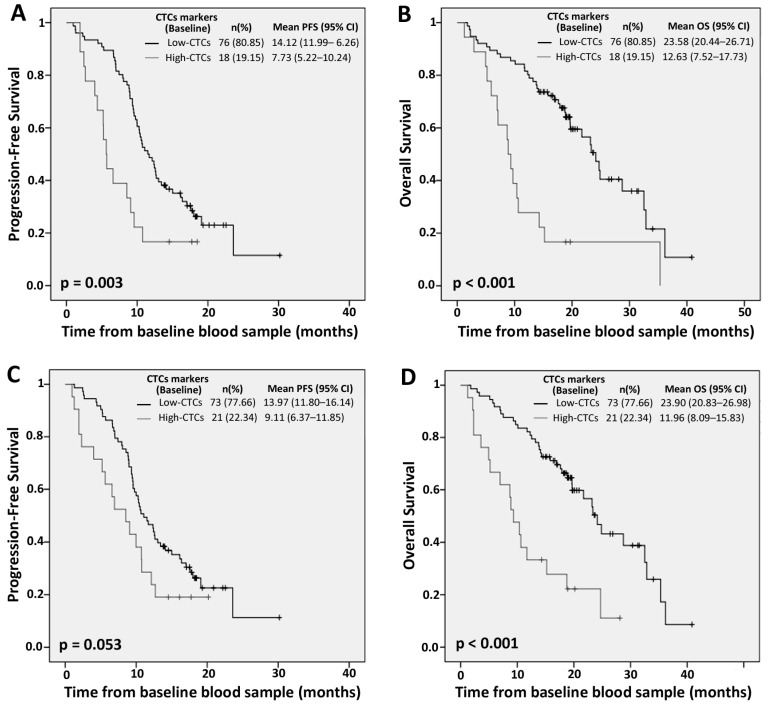
Kaplan-Meier plots of progression-free survival (PFS) and overall survival (OS) according to high or low PrediCTC expression levels at baseline (**A**,**B**) and four weeks after treatment onset (**C**,**D**).

Taking into account the expression levels of all seven markers together, each patient was assigned to the “low”- or “high”-CTCs groups, when at least four markers were below or above individual cut-offs, respectively. From the 94 mCRC patients analysed, 71 (75.53%) relapsed during follow-up, 15 of them (15.96%) being classified as high-CTCs by PrediCTC at baseline and significantly associated with lower PFS (14.12 vs. 7.73 months; *p* = 0.003) and shorter OS (23.57 vs. 12.62 months; *p* ≤ 0.001) rates (Figure 1). Similarly, OS rate was significantly shorter in patients classified as high-CTCs at the four week time point, after one chemotherapy cycle (23.90 vs. 11.96 months; *p* ≤ 0.001) (Figure 1). Univariate Cox regression analysis of clinical characteristics showed that the presence of lung metastasis and the baseline PrediCTC multimarker model were the only two independent prognostic factors for PFS and OS. Additionally, a significant correlation was found between the four-week follow-up PrediCTC model and the patients OS rate (Table 2).

These results confirmed the efficacy of the PrediCTC multimarker panel for the evaluation of the disseminated disease burden, demonstrating a prognostic value, both before treatment onset, and after one chemotherapy cycle.

### 2.3. Efficiency of PrediCTC for Clinical Outcome Assessment

**Figure 2:**
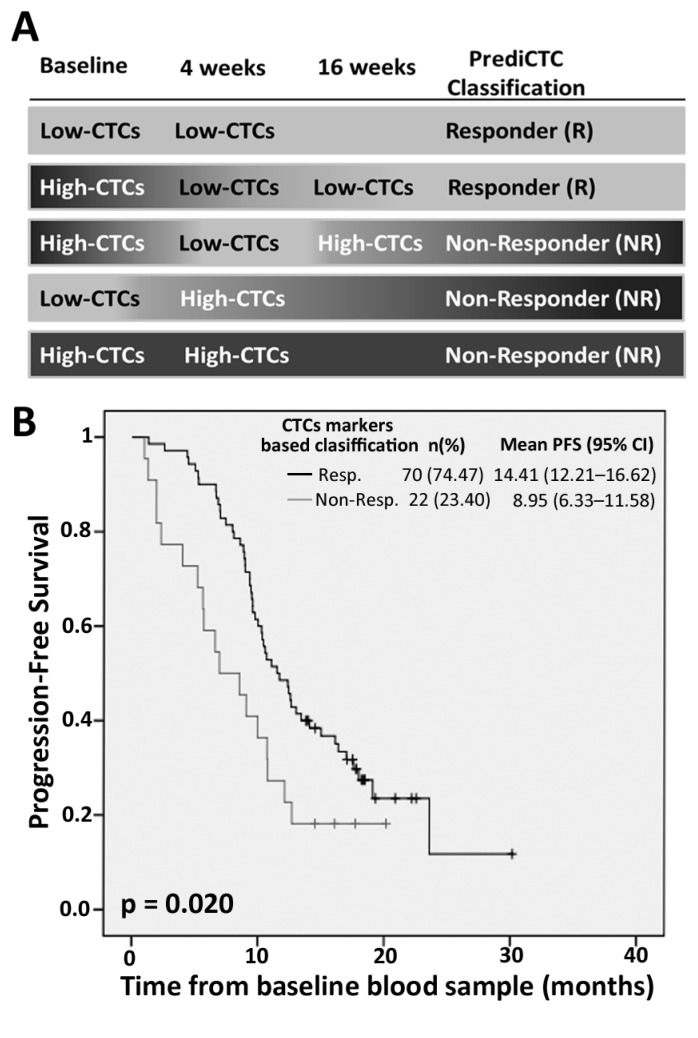

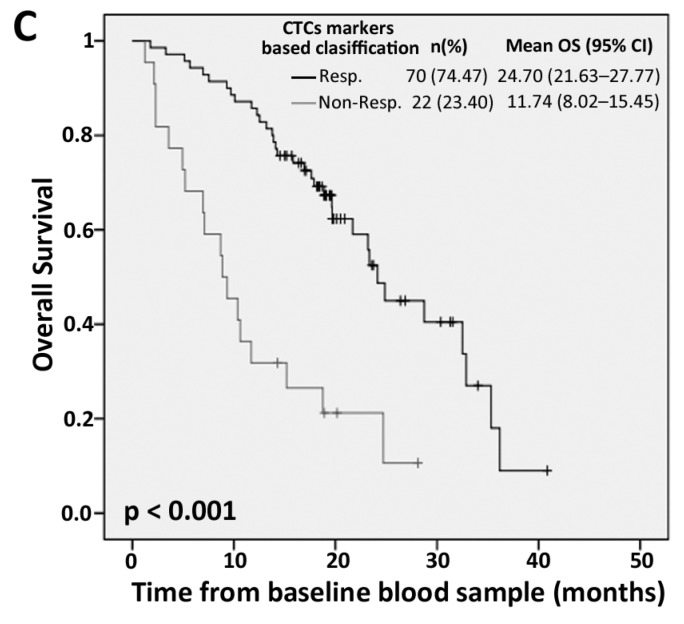
Kaplan-Meier curves showing progression-free survival (PFS) and overall survival (OS) based on the classification of patients as responders (Resp.) or non-responders (Non-Resp.) according to changes in circulating tumor cells (CTCs) gene expression levels with the treatment. (**A**) Classification of patients at the times of blood sample collection, according to PrediCTC. Kaplan-Meier curves for (**B**) PFS and (**C**) OS.

After confirming the prognostic value of PrediCTC, we aimed to test its efficiency for the classification of mCRC patients as responders or non-responders, as well as its predictive potential, by monitoring CTCs gene expression changes from baseline to the first chemotherapy cycle (four weeks). Patients were classified as schematized in Figure 2. Patients classified as high-CTCs by PrediCTC at both baseline and four-week time points, as well as patients presenting an increase in PrediCTC multimarker levels (e.g., switching from the low-CTCs group at baseline to high-CTCs at four-week follow-up) were classified as non-responders. Aversely, patients classified as low-CTCs by PrediCTC, both at baseline and at four-week time points were defined as responders. Finally, patients showing a decrease in marker expression levels from high-CTCs at baseline to low-CTCs after one cycle of chemotherapy were initially classified as Responders. However, for those patients, a third sample (after 16 weeks of treatment) was analyzed to confirm treatment response and to avoid false positives coming from fast responders with no overtime maintenance. Patients whose treatment response was not confirmed at the 16-week time point were reclassified as non-responders (see Scheme on Figure 2). Of note, only seven patients were subjected to a confirmatory sample collection at the 16 week time point, with only one patient being reclassified as non-responder. This means that 99% of patients analyzed in this work were classified as responders or non-responders after only one single cycle of chemotherapy.

Kaplan-Meier survival analysis showed that the mean PFS rate was 8.95 months for patients classified as non-responders (*n* = 22; 23.40%), significantly lower compared to the mean 14.41 months PFS for patients classified as responders (*n* = 70; 74.47%) (*p* = 0.020; Figure 2). In line with these findings, the mean OS rate was significantly reduced in patients classified as non-responders (11.74 months) compared to those classified as Responders (24.7 months) (*p* ≤ 0.001; Figure 2). Hazard ratios (HR) for the non-responder group (univariate Cox regression) was 1.88 for PFS (95% CI: 1.09–3.24, *p*=0.022) and 3.83 for OS (95% CI: 2.10–6.94, *p* < 0.001; Table 2). All variables that showed a significant prognostic value in the univariate analysis were independent predictors of worse PFS and OS in a multivariate analysis (Table 3).

These data confirmed our previous results obtained with a multimarker gene panel and validated PrediCTC as a predictive tool for the monitoring of therapy response in mCRC patients. Importantly, PrediCTC demonstrated efficacy after only one cycle of chemotherapy, earlier than routine imaging techniques (CT scan).

### 2.4. Competitive Advantage of PrediCTC versus Standard Computed Tomography (CT) Scan Monitoring

**Figure 3:**
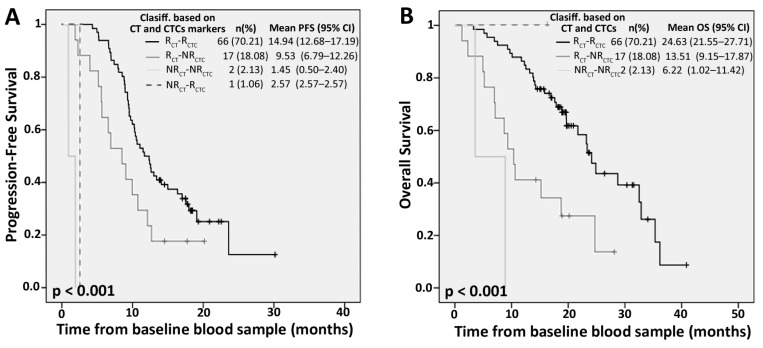
Kaplan-Meier curves showing progression-free survival (PFS) (**A**) and overall survival (OS) (**B**) according to the classification of a patient as responder (R) or non-responder (NR), based on the expression of the CTCs multimarker panel (CTC) and CT scan (CT).

In addition to efficiently assessing therapy response earlier than current standard monitoring techniques, we also evaluated the competitive advantage of PrediCTC to more reliably identify patients responding or not responding to therapy. As shown in Figure 3, 68 patients included in the study (72.34%) were equally classified by PrediCTC and CT scan, 66 patients (70.21%) as responders and 2 patients (2.13%) as non-responders. Mean PFS and OS rates for patients classified as responders by both techniques were significantly higher (PFS: 14.94, 95% CI: 12.68–17.19; OS: 24.63, 95% CI: 21.55–27.71) compared to those classified as non-responders (PFS: 1.45, 95% CI: 0.50–2.40; OS: 6.22 95% CI: 1.02–11.41) Interestingly, 17 patients (18.08%) were classified as non-responders by PrediCTC while they were defined as responders by CT scan. These patients evidenced a significantly reduced PFS and OS, compared with those defined as Responders by CT scan, demonstrating the improved ability of PrediCTC to identify those patients that are likely not responding to therapy (Figure 3). In addition, PrediCTC was also able to correctly classify one patient responding to therapy and identified as a non-responder by CT scan (Figure 3).

Overall, PrediCTC demonstrated an improved sensitivity for identification of therapy responders, after only one cycle of chemotherapy (four weeks), compared to a CT scan performed at the third cycle of therapy (12 weeks) (60% for PrediCTC versus 17.4% by CT scan). The specificity of the CT scan was 98.5%, compared to 89.6% with PrediCTC. This resulted in a PrediCTC Positive Predictive Value of 68.2%, defined as the probability of correctly classifying non-responders, and a negative predictive value of 85.7%, defined as the probability of correctly classifying responders. Consequently, PrediCTC drastically reduced the false negative rate from 82.6% as shown by CT scan, to 40%, meaning that PrediCTC is more efficient than CT scan at correctly identifying those patients that are not responding to therapy. In conclusion, PrediCTC demonstrated an improved global efficiency, defined as the probability of correctly classifying responders and non-responders among the whole population of false and real positive and negative patients, being 81.5% by PrediCTC versus 77.3% for the CT scan, in this multicentric study.

## 3. Discussion

Although the utility of CTCs in identifying patients with early stage disease who are at risk of developing recurrence, or as a tool to assess tumor therapy response is evident, the reality is that among all the approaches that have been developed for CTCs isolation and characterization, only the CellSearch System has been cleared by the FDA for its clinical use [19]. Using this system, the prognostic utility of CTCs detection has been recently evaluated in a meta-analysis composed of eleven studies, including 1847 patients with CRC [20]. The incidence of CTCs was significantly higher in the metastasis-positive group compared to the metastasis-negative group, as well as in the hepatic-metastasis-positive group. The presence of CTCs was significantly related to OS (HR = 2.00, 95% CI (1.49, 2.69), *p* < 0.01) and PFS (HR = 1.80, 95% CI (1.52, 2.13), *p* < 0.01), and for the CTCs positive group, the response rate both at baseline and during treatment, was significantly lower than for the negative group.

Despite its utility, the implementation of CTCs detection into the clinical practice remains a pending matter, and there exists a clear necessity to improve CTCs detection rate in mCRC disease with alternative or complementary technologies, to translate the use of CTCs as on-treatment biomarkers in future studies. For example, the analysis of CTCs, alone or in combination with CEA, may be a useful tool in monitoring the response to treatment in mCRC [21]. Also, the quantification of CTCs combining two methodologies, CellSearch and AdnaTest, demonstrated an improved detection of CTCs in a 47 mCRC patient cohort [22]. The requirement for improved sensitivity in CTCs detection has also been identified for the management of patients undergoing surgery [23]. We must also consider that the CTCs cohort in mCRC patients may be composed not only of epithelial tumor cells, but also tumor cells that have undergone transformation due to the epithelial-mesenchymal transition, and tumor stem cells [24]. Regarding to this fact, recent studies have demonstrated that, at the time of surgery, the detection of CTCs with CK20 by real-time polymerase chain reaction (RT-PCR) without using anti-EpCAM immunoisolation, is an independent prognostic factor for OS and DFS in colon cancer patients [25]. Likewise, the use of a complementary noninvasive tool in liquid biopsy, circulating tumor DNA (ctDNA), allows early diagnosis, prognosis and follow-up of response and resistance to therapy. In fact, the analysis of ctDNA in colorectal cancer patients may be a valuable biomarker that negatively correlates with survival [26]. Nevertheless, both CTCs and ctDNA-based liquid biopsies are promising tools for monitoring tumor dynamics, and should be standardized and validated in different independent trials, in order to be incorporated into standard clinical practice [27].

In this work, we presented a prospective multicentric study conducted in four hospitals of the public Galician health system, to validate the use of the PrediCTC marker panel in the prediction of outcome and therapy response in 94 mCRC patients. As shown, and in addition to its prognostic value, PrediCTC efficiently identified those patients that do not respond to first-line chemotherapy and presented reduced PFS and OS rates, after only one cycle of treatment. Moreover, for patient monitoring, PrediCTC relied only on a minimally invasive blood test, compared to sophisticated and complex standard CT scans. Finally, after only one chemotherapy cycle, PrediCTC demonstrated an improved efficiency in the evaluation of responses compared to CT scan imaging, which is performed at the third cycle of treatment (e.g., two months later than PrediCTC). This represents not only the elimination of two cycles of inefficacious therapy regimes that may cause undesirable secondary effects, but also reduces periods of uncertainty for patients and the oncologists regarding the assessment of response. In addition, to allow a rapid clinical decision when a therapy is not resultantly effective, PrediCTC might represent a saving in resources to the public health system. We are currently evaluating the cost-effectiveness of PrediCTC for its implantation in clinical routines.

## 4. Materials and Methods

### 4.1. Patients

A total of 94 mCRC patients, without prior treatment for metastatic disease were recruited at four hospitals in Spain (University Clinical Hospital of Santiago de Compostela, University Clinical Hospital of Ferrol, University Clinical Hospital of Ourense and University Clinical Hospital of Vigo). All patients signed an informed consent approved by the relevant ethical committee (RLL-ANT-2014-01; 17 October 2014; Rede Galega de Comités de Ética da Investigación). Inclusion criteria were: age ≥18 years, measurable mCRC (Stage IV), performance status of 0–2 based on the Eastern Cooperative Oncology Group (ECOG) scores, and the initiation of chemotherapy as first-line treatment. Patients with previous cancer diagnoses were not included in the study. Patient characteristics are summarized in Table 4. Metastasis location and the evolution of the disease were analyzed by a radiologist, using standard imaging techniques (CT scan) and following RECIST 1.1 guidelines [7]. Progressive disease was defined when there was at least a 20% increase in the sum of diameters of target lesions and/or the appearance of one or more new lesions. Patients dying from the disease during the follow-up period without being evaluated by CT scan were also considered as progression of disease. A volume of 7.5 mL of peripheral blood was collected from all patients at three different time points: just before initiation of treatment (baseline), after the first cycle of chemotherapy (four weeks) and before Cycle 5 (16 weeks).

### 4.2. CTCs Immunoisolation and Gene Expression Analysis

CTCs were isolated using CELLection Epithelial Enrich Dynabeads (Invitrogen, Dynal, Oslo, Norway) coated with monoclonal antibodies against human epithelial cell adhesion molecules (EpCAM), according to the user’s guide. RNA extraction and RT-qPCR were carried out as previously described [16]. mRNA isolation from CTCs samples was performed using the QiampViral kit (Qiagen, Valencia, CA, USA), optimized for very low cellularity samples, following manufacturer’s instructions. cDNA was synthesized using SuperScript III (Invitrogen, Carlsbad, CA, USA) and genes were specifically pre-amplified using the TaqMan^®^PreAmp Master Mix kit (Applied Biosystems, Foster City, CA, USA). Quality tests, data mining, and interpretation were performed as described [15,16,18].

mRNA levels of a panel of six genes (*GAPDH*, *VIL1*, *CLU*, *TIMP1*, *LOXL3* and *ZEB2*), previously identified in a global gene expression analysis of immunoisolated CTCs from mCRC patients [18], with the inclusion of the *TLN1* gene [16], were quantified in a StepOne plus thermocycler (Life Technologies, Carlsbad, CA, USA). TaqMan Gene Expression Assays (Applied Biosystems, Foster City, CA, USA) for selected genes (Table S2) were used, and sensitivity was demonstrated (Figure S1). As the analyzed genes are not exclusively expressed in CTCs but also in contaminating leucocytes, the expression levels of *PTPRC* (or *CD45*) were assessed to normalize marker expression levels against the background of unspecific immune-isolation; no differences were found for *CD45* expression during treatment (Figure S2). We already described the utility of *PTPRC* as a reference gene [16], as its expression levels are equal in mCRC patient samples and in healthy donors. All genes and appropriate positive and negative controls were measured in duplicate. Cq values (defined as the cycle number at which the fluorescence of a sample crosses a fixed threshold line) for each transcript were normalized to 40 (maximum number of cycles), and this value was then normalized to the 40-Cq value for *PTPRC* ((40-Cq target)-(40-Cq), to simplify interpretation of results. Accuracy of the methodology in terms of recovery rate (>90%), as well as non-interference of contaminating leukocytes, have been previously described [16].

### 4.3. Data evaluation and Statistical Analysis

Computed tomography was performed every 12 weeks from treatment onset. According to RECIST criteria, patients with stable disease, partial or complete response were classified as therapy responders, whereas patients with progressive disease were defined as non-responders. Progression-free survival (PFS) and overall survival (OS) rates were defined as the time (in months) between the first treatment day until disease progression or death, respectively.

Based on CTCs gene expression markers, patient with expression levels below or above the cutoff for 4 of the analyzed genes was included into low or high-CTCs groups, respectively. The cutoff value of each gene was determined according to a prior study [15] and established as the 75% percentile for each individual marker.

### 4.4. Statistical Analysis

To determine the association between progression-free survival or overall survival and gene markers levels, a Kaplan-Meier with log-rank test and univariate and multivariate COX regression survival analyses were used. A *p* value < 0.05 was considered statistically significant. Analyses were performed using SPSS 19.0 (SPSS Inc., Chicago, IL, USA).

## Figures and Tables

**Table 1:** Kaplan-Meier survival analysis of progression-free survival (PFS) and overall survival (OS), according to the expression of individual CTCs markers below (Low) or above (High) the cutoff at baseline and at four weeks follow-up.

Marker Levels	PFS			OS		
	Mean	95% CI	*p* Value	Mean	95% CI	*p* Value
Baseline						
*GAPDH*						
Low	14.05	11.83–16.26	0.052	22.93	19.61–26.26	**0.041**
High	9.40	6.84–11.96		17.30	11.79–22.81	
*VIL1*						
Low	14.15	11.94–16.63	**0.009**	23.60	20.41–26.79	**0.001**
High	8.69	6.43–10.95		13.90	10.11–17.69	
*CLU*						
Low	14.49	12.24–16.75	**0.001**	23.24	19.92–26.56	**0.014**
High	7.97	5.93–10.00		16.01	10.96–21.05	
*TIMP1*						
Low	14.24	12.09–16.39	**0.008**	24.32	21.07–27.58	**<0.001**
High	8.34	5.92–10.77		12.74	8.37–17.11	
*TLN1*						
Low	13.66	11.49–15.83	0.210	23.21	20.03–26.39	**0.004**
High	9.95	7.33–12.56		15.67	10.74–20.61	
*LOXL3*						
Low	14.30	11.97–16.63	**0.012**	23.10	19.78–26.43	**0.019**
High	8.94	6.77–11.11		16.76	11.45–22.06	
*ZEB2*						
Low	14.08	11.78–16.37	0.064	23.53	19.93–27.13	**0.009**
High	9.39	7.05–11.74		16.32	11.24–21.38	
Four-week follow-up						
*GAPDH*						
Low	14.03	11.91–16.15	**0.046**	24.05	21.02–27.07	**<0.001**
High	9.01	6.16–11.87		11.85	7.83–15.87	
*VIL1*						
Low	13.64	11.48–15.80	0.277	23.06	20.17–25.95	**0.006**
High	10.31	7.62–13.01		15.165	9.46–20.86	
*CLU*						
Low	13.66	11.47–15.84	0.241	22.80	19.90–25.70	**0.021**
High	10.63	7.69–13.57		16.38	10.45–22.31	
*TIMP1*						
Low	13.81	11.65–15.96	0.144	23.89	20.76–27.02	**<0.001**
High	9.62	6.96–12.28		12.83	9.07–16.59	
*TLN1*						
Low	13.66	11.57–15.75	0.268	23.74	20.66–26.82	**<0.001**
High	9.83	7.00–12.66		13.06	9.10–17.02	
*LOXL3*						
Low	14.24	12.05–16.43	**0.013**	23.75	20.64–26.86	**0.001**
High	8.53	6.01–11.05		14.31	9.06–19.57	
*ZEB2*						
Low	14.02	11.87–16.17	**0.049**	22.96	19.87–26.06	**0.022**
High	9.31	6.22–12.39		16.35	10.78–21.91	

CI: confidence interval. Data in bold indicates statistically significant *p* values (*p* ≤ 0.05).

**Table 2:** Untivariate Cox proportional hazard regression analysis for progression-free survival (PFS) and overall survival (OS) of patients’ clinical characteristics at baseline and the four-week follow-up PrediCTC multimarker model.

Covariate	*n*	PFS	*p* Value	OS	*p* Value
		HR (95% CI)		HR (95% CI)	
Age (≥66 vs. <66 years)	94	0.76 (0.48–1.22)	0.255	0.99 (0.58–1.69)	0.966
Sex (female vs. Male)	94	0.69 (0.42–1.14)	0.148	0.45 (0.24–0.87)	**0.017**
T stage (4 vs. ≤3)	64	0.86 (0.44–1.69)	0.667	0.84 (0.36–1.93)	0.678
N stage (2 vs. ≤1)	59	1.50 (0.85–2.66)	0.161	0.90 (0.44–1.87)	0.786
Hepatic mets. (yes vs. no)	91	0.76 (0.36–1.60)	0.474	0.98 (0.42–2.30)	0.964
Lung mets. (yes vs. no)	91	2.60 (1.54–4.38)	**<0.001**	2.45 (1.39–4.34)	**0.002**
Peritoneal mets. (yes vs. no)	91	1.27 (0.71–2.30)	0.422	1.53 (0.81–2.92)	0.192
Ganglionar mets. (yes vs. no)	91	1.26 (0.75–2.12)	0.391	1.00 (0.54–1.85)	0.996
No. of metastatic sites (≥2 vs. 1)	91	1.41 (0.88–2.26)	0.151	1.40 (0.80–2.44)	0.237
KRAS (mut. vs. WT)	91	0.92 (0.56–1.52)	0.754	1.07 (0.60–1.91)	0.815
Baseline CEA (≥10 vs. <10ng/mL)	91	1.27 (0.75–2.14)	0.379	1.03 (0.56–1.89)	0.918
ECOG PS (2 vs. ≤1)	94	1.51 (0.81–2.82)	0.199	1.91 (0.98–3.74)	0.058
Antibodies therapy (yes vs. no)	94	1.02 (0.64–1.63)	0.932	1.11 (0.65–1.90)	0.711
Baseline CTCs model (high-CTCs vs. low-CTCs)	94	2.33 (1.30–4.15)	**0.004**	3.37 (1.84–6.18)	**<0.001**
4 weeks CTCs model (high-CTCs vs. low-CTCs)	94	1.71 (0.99–2.95)	0.056	3.30 (1.82–5.99)	**<0.001**
CTCs model (NR vs. R)	92	1.88 (1.09–3.24)	**0.022**	3.83 (2.10–6.94)	**<0.001**

CTCs: circulating tumor cells; PFS: progression-free survival; OS: overall survival; HR: hazard ratio; CI: confidence interval; CEA: carcinoembrionic antigen; ECOG: Eastern Cooperative Oncology Group; PS: performance status; R: Responder; NR: Non-Responder; mets: metastasis; mut: mutated; WT: wild type. Bold font indicates statistically significant *p*-values (*p* ≤ 0.05).

**Table 3:** Multivariate Cox regression analysis for progression-free survival (PFS) and overall survival (OS) of patients’ clinical characteristics at baseline, and the four-week follow-up PrediCTC multimarker model.

Covariates	*n*	PFS	*p* Value	χ^2 (1)^	OS	*p* Value	χ^2 (1)^
		HR (95% CI)			HR (95% CI)		
Lung metastasis (yes vs. no)	91	2.82 (1.66–4.78)	**<0.001**	18.63	2.75 (1.54–4.90)	**0.001**	22.29
Baseline CTCs model (high-CTCs vs. low-CTCs)		2.42 (1.33–4.43)	**0.004**		3.66 (1.93–6.92)	**<0.001**	
Lung metastasis (yes vs. no)	91	2.81 (1.65–4.77)	**<0.001**	15.31	2.49 (1.41–4.15)	**0.002**	20.85
4 weeks CTCs model (high-CTCs vs. low-CTCs)		1.80 (1.02–3.19)	**0.043**		3.22 (1.74–5.95)	**<0.001**	
Lung metastasis (yes vs. no)	89	3.09 (1.80–5.30)	**<0.001**	18.50	2.76 (1.54–4.95)	**0.001**	25.80
CTCs model (NR vs. R)		2.06 (1.17–3.62)	**0.012**		3.82 (2.06–7.09)	**<0.001**	

CTCs: circulating tumor cells; PFS: progression-free survival; OS: overall survival; HR: hazard ratio; CI: confidence interval; R: Responder; NR: Non-Responder. Data in bold indicates statistical significance (*p* ≤ 0.05). ^(1)^ Significance of a χ-square test for multivariate models.

**Table 4:** Patient characteristics.

Characteristic	*n* (%)	Characteristic	*n* (%)
Gender		Metastasis location	
Male	59 (62.77)	Liver	38 (40.43)
Female	35 (37.23)	Liver and other	44 (46.81)
Primary tumor location		Nonliver	9 (9.57)
Colon	63 (67.02)	Unknown	3 (3.19)
Rectum	29 (30.85)	ECOG PS grade	
Both	2 (2.13)	0	10 (10.64)
KRAS status		1	70 (74.47)
Wild type	55 (58.51)	2	14 (14.89)
Mutated	36 (38.30)	First-line chemotherapy	
Unknown	3 (3.19)	Folfox	70 (74.47)
T		Folfiri	11 (11.70)
T_1_–T_2_	3 (3.19)	Capecitabine	5 (5.32)
T_3_	46 (48.94)	Capecitabine-Oxaliplatin	6 (6.38)
T_4_	15 (15.96)	Irinotecan	2 (2.13)
T_x_	30 (31.91)	First-line combined biological therapy	
N		Anti-EGFR	35 (37.23)
N_0_	10 (10.63)	Anti-VEGF	12 (12.76)
N_1_	24 (25.53)	None	47 (50)
N_2_	25 (26.59)		
N_3_	1 (1.06)		
N_x_	34 (36.17)		
Number of metastatic sites			
1	45 (47.88)		
≥2	46 (48.93)		
Unknown	3 (3.19)		

ECOG: Eastern Cooperative Oncology Group; PS: performance status; N: Nodal involvement; N_0_: no regional lymph nodes containing cancer cells; N_1_: metastasis in 1 to 3 lymph nodes; N_2_: metastasis into 4 or more lymph nodes; N_3_: metastasis into any nodes along the course of named vascular trunks; N_x_: unknown; EGFR: epidermal growth factor receptor; VEGF: vascular endothelial growth factor.

## Supplementary Materials

Supplementary materials can be found at www.mdpi.com/1422-0067/18/6/1265/s1.

## Abbreviations

CRC	Colorectal cancer
mCRC	Metastatic colorectal cancer
CT	Computed tomography
CTCs	Circulating tumor cells
PFS	Progression-free survival
OS	Overall survival
FDA	Food and Drug Administration
EMT	Epithelial to mesenchymal transition
CEA	Carcinoembryonic antigen
HR	Hazard ratio
CI	Confidence interval
R	Responder
NR	Non-responder
ECOG	Eastern Cooperative Oncology Group
PS	Performance status
Mests.	metastasis

## References

[1] Siegel R.L., Miller K.D., Fedewa S.A., Ahnen D.J., Meester R.G.S., Barzi A., Jemal A. (2017). Cancer statistics, 2017. CA. Cancer J. Clin..

[2] Van Cutsem E., Cervantes A., Nordlinger B., Arnold D., ESMO Guidelines Working Group (2014). Metastatic colorectal cancer: ESMO Clinical Practice Guidelines for diagnosis, treatment and follow-up. Ann. Oncol..

[3] Sobrero A.F., Maurel J., Fehrenbacher L., Scheithauer W., Abubakr Y.A., Lutz M.P., Vega-Villegas M.E., Eng C., Steinhauer E.U., Prausova J. (2008). EPIC: Phase III trial of cetuximab plus irinotecan after fluoropyrimidine and oxaliplatin failure in patients with metastatic colorectal cancer. J. Clin. Oncol..

[4] Cassidy J., Clarke S., Díaz-Rubio E., Scheithauer W., Figer A., Wong R., Koski S., Lichinitser M., Yang T.-S., Rivera F. (2008). Randomized phase III study of capecitabine plus oxaliplatin compared with fluorouracil/folinic acid plus oxaliplatin as first-line therapy for metastatic colorectal cancer. J. Clin. Oncol..

[5] Stathopoulos G.P., Batziou C., Trafalis D., Koutantos J., Batzios S., Stathopoulos J., Legakis J., Armakolas A. (2010). Treatment of colorectal cancer with and without bevacizumab: A phase III study. Oncology.

[6] Passardi A., Nanni O., Tassinari D., Turci D., Cavanna L., Fontana A., Ruscelli S., Mucciarini C., Lorusso V., Ragazzini A. (2015). Effectiveness of bevacizumab added to standard chemotherapy in metastatic colorectal cancer: Final results for first-line treatment from the ITACa randomized clinical trial. Ann. Oncol. Off. J. Eur. Soc. Med. Oncol..

[7] Eisenhauer E.A., Therasse P., Bogaerts J., Schwartz L.H., Sargent D., Ford R., Dancey J., Arbuck S., Gwyther S., Mooney M. (2009). New response evaluation criteria in solid tumors: Revised RECIST guideline (version 1.1). Eur. J. Cancer.

[8] Van Cutsem E., Verheul H.M.W., Flamen P., Rougier P., Beets-Tan R., Glynne-Jones R., Seufferlein T. (2016). Imaging in colorectal cancer: Progress and challenges for the clinicians. Cancers.

[9] Nguyen D.X., Bos P.D., Massagué J. (2009). Metastasis: From dissemination to organ-specific colonization. Nat. Rev. Cancer.

[10] Cohen S.J., Punt C.J.A., Iannotti N., Saidman B.H., Sabbath K.D., Gabrail N.Y., Picus J., Morse M., Mitchell E., Miller M.C. (2008). Relationship of circulating tumor cells to tumor response, progression-free survival, and overall survival in patients with metastatic colorectal cancer. J. Clin. Oncol..

[11] Cohen S.J., Punt C.J.A., Iannotti N., Saidman B.H., Sabbath K.D., Gabrail N.Y., Picus J., Morse M.A., Mitchell E., Miller M.C. (2009). Prognostic significance of circulating tumor cells in patients with metastatic colorectal cancer. Ann. Oncol..

[12] Hayes D.F., Cristofanilli M., Budd G.T., Ellis M.J., Stopeck A., Miller M.C., Matera J., Allard W.J., Doyle G.V, Terstappen L.W.W.M. (2006). Circulating tumor cells at each follow-up time point during therapy of metastatic breast cancer patients predict progression-free and overall survival. Clin. Cancer Res..

[13] Hartkopf A.D., Wagner P., Wallwiener D., Fehm T., Rothmund R. (2011). Changing levels of circulating tumor cells in monitoring chemotherapy response in patients with metastatic breast cancer. Anticancer Res..

[14] De Bono J.S., Scher H.I., Montgomery R.B., Parker C., Miller M.C., Tissing H., Doyle G.V., Terstappen L.W., Pienta K.J., Raghavan D. (2008). Circulating tumor cells predict survival benefit from treatment in metastatic castration-resistant prostate cancer. Clin Cancer Res..

[15] Barbazán J., Muinelo-Romay L., Vieito M., Candamio S., Díaz-López A., Cano A., Gómez-Tato A., de los Ángeles Casares de Cal M., Abal M., López-López R. (2014). A multimarker panel for circulating tumor cells detection predicts patient outcome and therapy response in metastatic colorectal cancer. Int. J. Cancer.

[16] Barbazán J., Alonso-Alconada L., Muinelo-Romay L., Vieito M., Abalo A., Alonso-Nocelo M., Candamio S., Gallardo E., Fernández B., Abdulkader I. (2012). Molecular characterization of circulating tumor cells in human metastatic colorectal cancer. PLoS ONE.

[17] Barbazán J., Alonso-Alconada L., Elkhatib N., Geraldo S., Gurchenkov V., Glentis A., van Niel G., Palmulli R., Fernandez B., Viaño P. (2017). Liver metastasis is facilitated by the adherence of circulating tumor cells to vascular fibronectin deposits. Cancer Res..

[18] Barbazán J., Vieito M., Abalo A., Alonso-Alconada L., Muinelo-Romay L., Alonso-Nocelo M., León L., Candamio S., Gallardo E., Anido U. (2012). A logistic model for the detection of circulating tumor cells in human metastatic colorectal cancer. J. Cell. Mol. Med..

[19] Hardingham J.E., Grover P., Winter M., Hewett P.J., Price T.J., Thierry B. (2015). Detection and clinical significance of circulating tumor cells in colorectal cancer--20 years of progress. Mol. Med..

[20] Huang X., Gao P., Song Y., Sun J., Chen X., Zhao J., Xu H., Wang Z. (2015). Meta-analysis of the prognostic value of circulating tumor cells detected with the CellSearch System in colorectal cancer. BMC Cancer.

[21] Das A., Kunkel M., Joudeh J., Dicker D.T., Scicchitano A., Allen J.E., Sarwani N., Yang Z., Kaifi J., Zhu J. (2015). Clinico-pathological correlation of serial measurement of circulating tumor cells in 24 metastatic colorectal cancer patients receiving chemotherapy reveals interpatient heterogeneity correlated with CEA levels but independent of KRAS and BRAF mutation. Cancer Biol. Ther..

[22] Gorges T.M., Stein A., Quidde J., Hauch S., Röck K., Riethdorf S., Joosse S.A., Pantel K. (2016). Improved detection of circulating tumor cells in metastatic colorectal cancer by the combination of the CellSearch® System and the AdnaTest^®^. PLoS ONE.

[23] Vojtechova G., Benesova L., Belsanova B., Minarikova P., Levy M., Lipska L., Suchanek S., Zavoral M., Minarik M. (2016). Monitoring of circulating tumor cells by a combination of immunomagnetic enrichment and RT-PCR in colorectal cancer patients undergoing surgery. Adv. Clin. Exp. Med..

[24] Gorges T.M., Tinhofer I., Drosch M., Röse L., Zollner T.M., Krahn T., von Ahsen O. (2012). Circulating tumor cells escape from EpCAM-based detection due to epithelial-to-mesenchymal transition. BMC Cancer.

[25] Hinz S., Hendricks A., Witting A., Schafmayer C., Tepel J., Kalthoff A., Becker T., Röder C. (2017). Detection of circulating tumor cells with CK20 RT-PCR is an independent negative prognostic marker in colon cancer patients—A prospective study. BMC Cancer.

[26] Fan G., Zhang K., Yang X., Ding J., Wang Z., Li J. (2017). Prognostic value of circulating tumor DNA in patients with colon cancer: Systematic review. PLoS ONE.

[27] Tan C.R.C., Zhou L., El-Deiry W.S. (2016). Circulating tumor cells versus circulating tumor DNA in colorectal cancer: Pros and cons. Curr. Colorectal Cancer Rep..

