# Supplementary material for: Predicting Outcome and Therapy Response in mCRC Patients Using an Indirect Method for CTCs Detection by a Multigene Expression Panel: A Multicentric Prospective Validation Study
Source: Int J Mol Sci. 2017 Jun 13;18(6):1265. doi: 10.3390/ijms18061265 (PMC5486087; doi:10.3390/ijms18061265)

Table S1. Relative gene expression levels of CTCs markers (*PTPRC* normalized) and percentiles 25% and 75%.

	Baseline					
Gene	Mean	Median	SD	Mean	Median	SD
GAPDH	3.40	3.34	1.64	4.12	3.87	1.65
VIL1	-4.27	-4.06	2.96	-2.95	-3.11	2.96
CLU	4.16	4.20	2.56	5.11	5.03	2.83
TIMP1	1.97	1.76	2.03	3.06	2.76	1.96
TLN1	3.33	3.28	1.93	4.18	3.86	2.20
LOXL3	-3.54	-3.53	2.18	-3.00	-3.25	2.18
ZEB2	0.64	0.47	1.30	1.16	0.99	1.61

	75% Q		
Gene	B	4-W	16-W
GAPDH	4.45	4.77	4.99
VIL1	-2.65	-1.53	-1.37
CLU	5.84	6.56	6.51
TIMP1	3.12	3.85	4.09
TLN1	4.39	5.23	5.27
LOXL3	-2.13	-1.85	-1.74
ZEB2	1.53	1.70	2.02

Abbreviations: B, Baseline; 4-W, 4 Weeks; 16-W, 16 Weeks; Q, Quartile; SD, Standard deviation.

Table S2. References of the RTqPCR TaqMan probes of the multimarker panel.

**Gene Name**	**TaqMan Probe Reference**
PTPRC	Hs00894734_m1
GAPDH	Hs99999905_m1
VIL1	Hs00200229_m1
TIMP1	Hs00171558_m1
CLU	Hs00156548_m1
LOXL3	Hs01046945_m1
ZEB2	Hs00207691_m1
TLN1	Hs00196775_m1

Figure S1. Accuracy of *GAPDH* normalized to *CD45* in CTCs detection. Bars represent 40-Cq values of *GAPDH* normalized to 40-*CD45* of a growing number of HCT116 colon cancer tumor cells subjected to immunoisolation in 7.5 ml of blood. Results from 3 different assays.

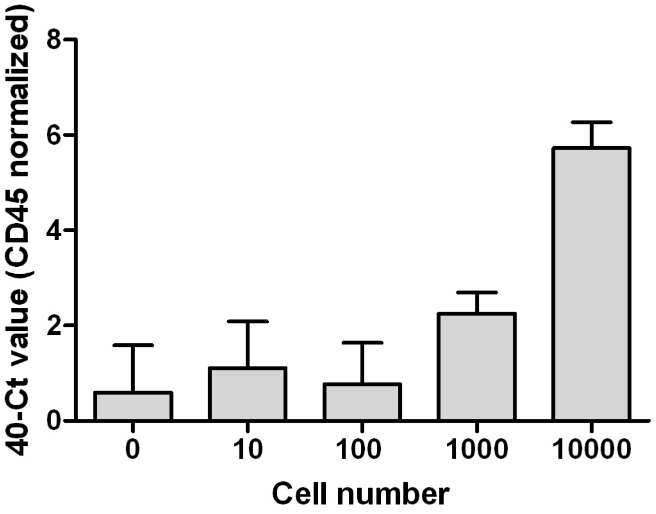

Figure S2. Box plots indicate median values of *CD45* expression of patient cohorts over time.

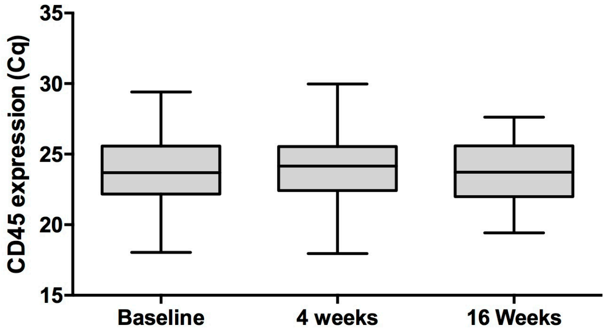

Supplement: Supplementary file 1 [file ijms-18-01265-s001.pdf]
